# Intraindividual Changes in Rheumatoid Factor and Anti-Cyclic Citrullinated Peptide Antibody Tests in Korean Patients Visiting Local Clinics and Hospitals

**DOI:** 10.3390/jcm11030832

**Published:** 2022-02-04

**Authors:** Rihwa Choi, Sang Gon Lee, Eun Hee Lee

**Affiliations:** 1 Department of Laboratory Medicine, Green Cross Laboratories, Yongin 16924, Korea; pirate0720@naver.com; 2 Department of Laboratory Medicine and Genetics, Samsung Medical Center, Sungkyunkwan University School of Medicine, Seoul 06351, Korea; 3 Green Cross Laboratories, Yongin 16924, Korea

**Keywords:** rheumatoid arthritis, rheumatoid factor, anti-cyclic citrullinated peptide antibodies, test utilization, intraindividual change, Korea

## Abstract

Limited data are available on test utilization and intraindividual changes in rheumatoid factor (RF) and anti-cyclic citrullinated peptide antibody (anti-CCP) in Korean patients that visit local clinics and hospitals. We retrospectively reviewed longitudinally measured RF and anti-CCP data in Korean patients to investigate the utilization and changes in test results through a laboratory information system. During the 10-year study period, 256,259 specimens were tested for RF. Among them, 32,567 (12.7%) specimens from 31,110 Korean adults had simultaneously measured anti-CCP results. Among them, 1110 (3.6%) subjects had follow-up test results. Among 351 patients with initial positive RF results, 290 (82.6%) had no qualitative change in RF from positive to negative values during follow-up. About 3.8% (29/759) of patients with initial negative results experienced qualitative changes in RF that were positive on follow-up. Among 182 patients with an anti-CCP-positive result at initial measurement, 174 (95.6%) had no qualitative change in anti-CCP from positive to negative or equivocal results during follow-up. About 0.5% (5/928) of patients with initial negative values experienced qualitative changes in anti-CCP to positive values on follow-up. The agreement of qualitative results between RF and anti-CCP was 80.8% (95% confidence interval 78.4–83.1%) at initial measurement and 80.6% (95% confidence interval 79.0–82.1%) overall. The results of this study can help inform utilization of RF and anti-CCP testing for Korean patients visiting local clinics and hospitals.

## 1. Introduction

Rheumatoid arthritis (RA) is a chronic inflammatory autoimmune joint disease that is pathologically heterogeneous [1]. It has been reported that the presence of autoantibodies, such as rheumatoid factor (RF) and anti-cyclic citrullinated peptide antibody (anti-CCP), is associated with more severe symptoms and joint damage, as well as increased mortality [2]. Previous studies have reported that the presence of anti-CCP and RF is associated with a more aggressive disease course, and these factors have been suggested for use as both diagnostic and prognostic markers [3,4,5]. Although there are no confirmed diagnostic criteria for RA, early diagnosis is important for improving patient outcomes, and the classification criteria proposed by the American College of Rheumatology/European League Against Rheumatism (ACR/EULAR) include clinical and serological variables such as RF and anti-CCP, which can aid in diagnosing RA [2]. The ACR/EULAR criteria also propose that, if RF assay results are only available qualitatively, a positive result should be scored as a low-positive [4,6]. Further, with this testing approach and based on local laboratory standards, values equal to or less than the upper limit of normal (ULN) for the respective laboratory are defined as “Negative for RF” and are assigned 0 points; values that are >1–3 times the ULN are defined as “Low-positive” and assigned 2 points, and values that are >3 times the ULN are defined as “High-positive” and are assigned 3 points, based on ACR/EULAR 2010 classification criteria suggesting the importance of autoimmune serology in RA classification [4,5]. Recent studies have reported that anti-CCP can be detected up to 10 years before diagnosis, which has been referred to as preclinical RA [1,4,7].

The stability and fluctuation of RF and anti-CCP levels throughout the disease course have been studied and the significance of the change in RF and anti-CCP remains unclear [1,8]. Some studies have reported that the levels are stable during the disease course, while others have found that both RF and anti-CCP concentrations decrease with effective therapy [1,8]. However, patients rarely become anti-CCP-negative, whereas RF decreases more profoundly and more frequently and patients can seroconvert and become RF negative [1,9]. Some reports have indicated that the level of RF decreases more strongly than does the anti-CCP level, suggesting a greater plasticity or a different cellular origin of RF with effective treatment [4]. However, the optimal clinical use of anti-CCP and RF tests is unclear [1,4,10,11,12].

Although reports have indicated that RF and anti-CCP tests can be used in autoimmune diseases in Korean populations, most of those studies were performed using populations visiting university hospitals. Only limited data are available on the utilization of RF and anti-CCP in local clinics and hospitals in Korea, and intraindividual quantitative or qualitative changes of those serologic markers were measured longitudinally in those Korean patients. Therefore, in this study, we retrospectively investigated the test utilization of RF and anti-CCP and intraindividual changes of longitudinally measured RF and anti-CCP results in Korean patients visiting local clinics and hospitals. Furthermore, we aimed to investigate the concurrence between RF and anti-CCP levels and the corresponding RF value for predicting anti-CCP positivity. This study may bridge the knowledge gaps between patient populations visiting local clinics and hospitals and those visiting university hospitals and tertiary medical centers.

## 2. Materials and Methods

**Figure 1:**
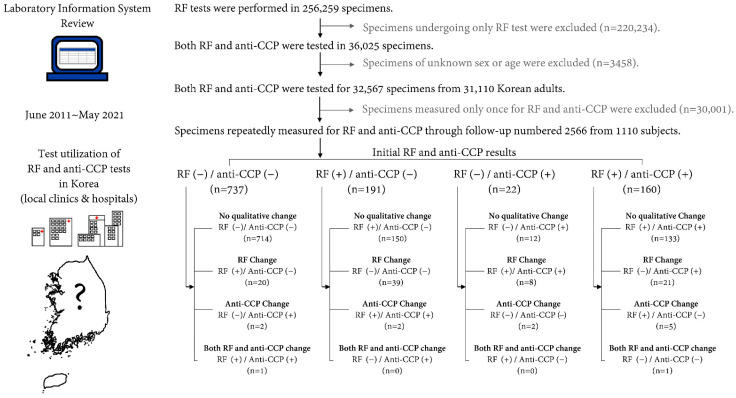
Flow diagram for this study in accordance with rheumatoid factor (RF) and anti-cyclic citrullinated peptide antibody (anti-CCP) test results. For assessment of qualitative change of anti-CCP results, equivocal and negative test results were considered “not positive” and presented as (−) in the diagram. Quantitative results of RF ≥ 14 IU/mL and anti-CCP > 10 U/mL were defined as “positive”.

We retrospectively reviewed data obtained through the laboratory information system from Korean adult (>19 years) patients who underwent serum RF tests and/or anti-CCP tests between June 2011 and May 2021, by Green Cross Laboratories. Among them, results were excluded for (1) patients with missing data for age or sex and (2) patients that only underwent RF and anti-CCP tests once during the study period. All data were anonymized prior to statistical analysis. The flow diagram for this study is presented in Figure 1. This study was conducted according to the guidelines outlined in the Declaration of Helsinki, and all procedures involving human subjects were approved by the Institutional Review Board of Green Cross Laboratories (GCL-2021-1044-01).

Because the anti-CCP test has been reimbursed since 1 September 2020 in Korea, nationwide utilization of anti-CCP testing is available from Healthcare Bigdata Hub by Health Insurance Review & Assessment Service (HIRA) in Korea (https://opendata.hira.or.kr/, accessed on 28 December 2021), with the test code D8130. Between 1 September 2020 and 31 May 2021 (the end of the stud period), 361,274 anti-CCP tests were performed in 341,819 Korean subjects. However, the number of subjects who underwent both RF and anti-CCP tests was not available in the Healthcare Bigdata Hub by HIRA. Green Cross Laboratories is one of the biggest referral clinical laboratories in Korea, providing clinical services for both RF and anti-CCP tests throughout Korea. In this study period, anti-CCP tests were performed in 20,046 Korean subjects in GCLabs (about 5.9% of 341,819 Koreans who underwent anti-CCP testing during the same period).

Serum RF tests were performed using a Tina-quant RF II assay kit on Modular P analyzers (Roche Diagnostics, Mannheim, Germany) from 1 June 2011 to 6 November 2016 and on a Rheumatoid Factors II assay kit on a Cobas 8000 platform (Cobas c702 analyzers, Roche Diagnostics, Mannheim, Germany) from 7 November 2016 to 31 May 2021 according to the manufacturer’s instructions. The cutoff for determination of RF was 14 IU/mL, as suggested by the manufacturer. Quantitative results of RF ≥ 14 IU/mL were defined as “positive” for qualitative interpretation. Anti-CCP tests were performed using an EliA CCP assay kit on ImmunoCAP 250 analyzers (Phadia AB, Uppsala, Sweden). The EliA CCP assay kit uses citrullinated synthetic peptides (second-generation antigen). For qualitative interpretation of anti-CCP, samples with <7.0 U/mL were defined as “negative”, samples with 7.0–10.0 U/mL were defined as “equivocal”, and samples with >10.0 U/mL were defined as “positive”, according to the manufacturer’s instruction. Analytical methods for the anti-CCP test in the laboratory did not change during the study period. The accuracy of RF and anti-CCP tests has been confirmed by participation in the proficiency testing program by the College of American Pathologists.

Chi-square tests were used to compare results for categorical variables (qualitative results of RF and anti-CCP) and Mann–Whitney tests were used when appropriate to compare results for continuous variables (age and quantitative value of RF and anti-CCP). Anti-CCP test results were classified as a binary result of positive (>10.0 U/mL) or not positive to investigate the agreement between qualitative results for RF and anti-CCP [13] and to investigate the qualitative changes of test results. Statistical analysis was executed using MedCalc software for Windows, version 19.1.5 (MedCalc Software bvba, Ostend, Belgium). A *p*-value < 0.05 was considered statistically significant.

## 3. Results

During the 10-year study period, 256,259 specimens were tested for RF. Among them, 32,567 (12.7%) specimens from 31,110 Korean adults (11,358 men and 19,752 women) with a median age of 50.3 years (interquartile range 38.8 to 58.9 years) had simultaneously measured anti-CCP results. Among 31,110 Korean adults with both RF and anti-CCP results, 2566 test results from 1110 (3.6%) subjects (392 men and 718 women) included in this study. The median (interquartile range) number of follow-up measurements was 2 (2–3 times) with a median (interquartile range) follow-up period of 11.0 (5.2–23.3) months. Basal characteristics of the study subjects are summarized in Table 1.

**Figure 2:**
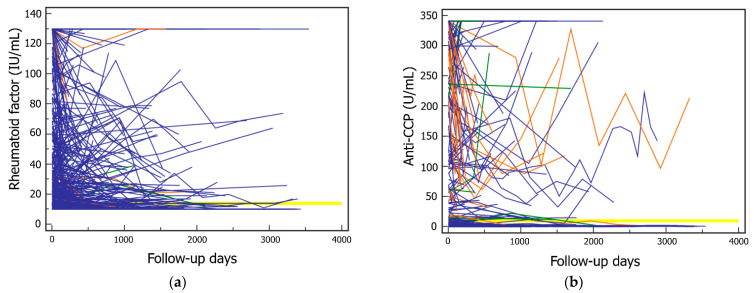
Intraindividual changes in rheumatoid factor (RF) and anti-cyclic citrullinated peptide antibody (anti-CCP): (**a**) changes in RF level by group and qualitative change in anti-CCP (blue: no qualitative change in anti-CCP in 1097 subjects, green: initial negative changed to positive value for anti-CCP in 5 subjects, orange: initial positive changed to negative for anti-CCP in 8 subjects); (**b**) changes in anti-CCP level by group for qualitative change in RF (blue: no qualitative change in RF in 1020 subjects, green: initial negative changed to positive for RF in 29 subjects, orange: initial positive changed to negative for RF in 61 subjects). Horizontal yellow lines indicate the upper limit of the reference interval (cutoff for RF: 14 IU/mL, cutoff for anti-CCP: 10 U/mL).

Among 1110 subjects having both RF and anti-CCP test results, 759 (68.4%) had RF negative results at initial measurement. Among these, 730 (96.2%) had no qualitative change in RF from negative to positive during follow-up. Among 351 patients that were initially RF-positive, 290 (82.6%) had no qualitative change in RF from positive to negative during follow-up. About 3.8% (29) of patients with initial negative results experienced qualitative changes in RF to positive. Overall, the rate of subjects with stable RF negative or positive results during follow-up was 93.5%. Among 1110 subjects, 928 (83.6%) had anti-CCP-negative or equivocal results at initial measurement. Among these, 923 (99.5%) had no qualitative change in anti-CCP from negative or equivocal to positive during follow-up. About 0.5% of patients (5 patients) with initial negative results experienced qualitative changes in anti-CCP to positive. Among 182 patients with anti-CCP-positive results at initial measurement, 174 (95.6%) had no qualitative change in anti-CCP from positive to negative or equivocal result during follow-up. Overall, the rate of subjects with stable anti-CCP results during follow-up was 98.8%. Intraindividual changes in RF and anti-CCP are shown in Figure 2. The maximum difference in the quantitative levels of RF and ant-CCP between initial and follow-up measurement ranged from −112 to 105 IU/mL for RF and from −270 to 230 U/mL for anti-CCP.

The agreement between RF and anti-CCP results is summarized in Table 2. The overall percentage agreement of 1110 test results measured initially between RF and anti-CCP was 80.8% (95% confidence interval 78.4–83.1%), the positive percentage agreement was 45.6% (95% confidence interval 40.3–51.0%), and the negative percentage agreement was 97.1% (95% confidence interval 95.6–98.2%). The overall percentage agreement of all 2566 test results between RF and anti-CCP during the study period was 80.6% (95% confidence interval 79.0–82.1%), the positive percentage agreement was 48.5% (95% confidence interval 45.1–52.0%), and the negative percentage agreement was 96.2% (95% confidence interval 95.2–97.1%).

## 4. Discussion

In this study, we retrospectively investigated test utilization and intraindividual changes in RF and anti-CCP in Korean patients who visited local clinics and hospitals. According to data from Healthcare Bigdata Hub by HIRA in Korea, about half of patients with RA, unspecified (the Korean Classification of Disease (KCD) M06: RA, unspecified), visited local clinics and hospitals rather than university hospitals and medical centers. Meanwhile, only about 20% of all RA patients with RF (KCD M05: RA with RF) visited local clinics and hospitals. There are knowledge gaps about Korean RA patients due to differences in characteristics of patients visiting local clinics and hospitals in comparison with those visiting university hospitals and medical centers. This study may play a role in bridging these knowledge gaps.

The inconsistency of qualitative results between RF and anti-CCP is a well-known issue in RA patients [1,4]. In the present study, the overall agreement between RF and anti-CCP was about 80%. Because RF is associated with a low specificity for diagnosing RA, anti-CCP has been introduced as a useful biomarker for diagnosing RA, based on current recommendations [6]. Although there are no diagnostic criteria for RA, early diagnosis can be critical for improving patient outcomes, and the classification criteria proposed by the ACR/EULAR include RF and anti-CCP for classifying RA patients [2,6].

Disease progression of RA involves the initiation and propagation of autoimmunity [4]. Preclinical RA (asymptomatic and early symptomatic autoimmunity) with increased levels of autoantibodies and inflammatory cytokines, chemokines, and C-reactive protein can progress to undifferentiated arthritis (early RA) and classifiable RA with expansion of the autoantibody profile, resulting in detectable RF and anti-CCP up to 10 years before clinical disease onset [4]. It has been reported that individuals who have both RF and anti-CCP with increased C-reactive protein develop symptoms more rapidly (within a few months) than those who have only anti-CCP [1].

Current monitoring approaches for patients with RA include inflammatory biomarkers such as C-reactive protein and erythrocyte sedimentation rate, both of which are used as laboratory tests [4,6]. However, the significance of changes in anti-CCP as well as the coexistence of RF and anti-CCP during the disease course and management remains unclear [3,14,15,16,17,18].

Previous studies on RA patients that were recruited based on clinical symptoms reported no significant prognostic value for RF or anti-CCP [5], while other studies reported that patients with high levels of anti-CCP were more likely to relapse from remission [15]. A study performed in Japan reported that fluctuation in anti-CCP level showed a weak association with disease activity using Disease Activity Score 28-joint count (DAS28) with ESR, clinical disease activity index (CDAI), and a simplified disease activity index (SDAI) and predicted relapse from remission in patients with RA [15]. Recent studies reported that RF- and anti-CCP-positive patients were associated with extra-articular manifestations, such as systemic vasculitis, pericarditis, and RA-related interstitial lung disease (RA-ILD) [5,16,17]. Future studies to investigate the clinical significance of fluctuations in RF and anti-CCP are needed.

Recent studies focused on the use of RF and anti-CCP for prediction of outcome in response to therapy, such as tumor necrosis factor α inhibitors, reported associations of these antibodies and biologic disease-modifying antirheumatic drugs [5]. In this vein, the 2019 update of the EULAR RA management recommendations includes an assessment of both RF and anti-CCP as prognostic factors to escalate the therapeutic treatment options such as the biologic disease-modifying anti-rheumatic drug of Janus kinase inhibitors during treatment and monitoring in patients with RA [19]. Current clinical practice guideline on the use of biological disease-modifying antirheumatic drugs for inflammatory arthritis in Korea includes RF and anti-CCP as prognostic factors [20]. Recent clinical practice guidelines on RA treatment by the ACR do not include prognostic factors such as RF and anti-CCP [21].

In the present study, 12.7% of RF tests had concurrently measured anti-CCP, and only 3.6% of those patients were followed up for both RF and anti-CCP measurements. In South Korea, anti-CCP testing is reimbursable partially or totally by HIRA, since 1 September 2020. The reimbursement condition can affect the utilization of anti-CCP tests that are needed for escalated treatment with other therapeutic options [22]. In the present study, some patients experienced qualitative changes in RF and anti-CCP values, and these patients might need options for changes in treatment. Therefore, future studies about the clinical significance and outcomes of RF and anti-CCP tests and the impact of their utilization are needed in large numbers of patients to improve patient outcomes.

There are some limitations to this study. One was lack of clinical information, including detailed history; physical examination; imaging findings; other laboratory measures associated with autoimmune diseases, such as other autoimmune antibodies, C-reactive protein, and erythrocyte sedimentation rate; serologic markers for cytokine activity and inflammation; and information on any medication, especially on B-cell-depleting therapies. Low absolute numbers of subjects who experienced qualitative changes in RF and anti-CCP in the present study might be due to the insufficient follow-up period to identify such qualitative changes. RF and anti-CCP can be measured and followed for development of autoimmune diseases other than RA. The results might not be generalizable to other ethnic populations since this study only included Korean adults. Because RF and anti-CCP tests are not globally well standardized, the citrullinated synthetic peptide (second-generation antigen) reagent to identify anti-CCP might affect the generalizability [5]. Recent studies reported additive values for improving diagnostic accuracy using a third-generation anti-CCP assay in addition to the second-generation assay to diagnose RA [5,23]. However, one strength of this study was the inclusion of population data from patients visiting local clinics and hospitals in Korea. Previous studies performed in Korea usually represent patients visiting university hospitals and medical centers. This study using longitudinally measured RF and anti-CCP results from patients visiting local clinics in Korea will help to fill knowledge gaps on intraindividual changes in Korean patients visiting local clinics. The present study represents a starting point for further investigations including clinical information. Future studies with larger numbers of study subjects and detailed clinical findings are needed to assess the clinical implications of changes in RF and anti-CCP.

## 5. Conclusions

In conclusion, we evaluated the test utilization of and intraindividual changes in RF and anti-CCP in Korean patients visiting local clinics and hospitals. Some patients had qualitative changes in RF and anti-CCP during follow-up. Future studies to clarify the clinical implications of RF and anti-CCP are needed to further understand and inform appropriate utilization to improve patient outcomes.

## Figures and Tables

**Table 1:** Baseline characteristics of 1110 Korean subjects (initial test results).

Characteristics	Total (*n* = 1110)		Men (*n* = 392)		Women (*n* = 718)	
	Median	IQR	Median	IQR	Median	IQR
Age (years)	53.1	42.2 to 61.8	49.4	35.7 to 58.8	54.5	46.2 to 62.7
Rheumatoid factor (IU/mL)	10.0	10.0 to 19.0	10.0	10.0 to 12.5	10.0	10.0 to 23.0
Anti-CCP (U/mL)	1.2	0.8 to 2.0	1.1	0.7 to 1.6	1.3	0.8 to 2.3
**Qualitative test results of initial measure, number of positive (%)**						
Rheumatoid factor (≥14 IU/mL)	351	(31.6%)	90	(20.3%)	261	(36.4%)
Anti-CCP (>10 U/mL)	182	(16.4%)	45	(11.5%)	137	(19.1%)

Data from patients with follow-up samples. Abbreviations: anti-CCP, anti-cyclic citrullinated peptide antibody; IQR, interquartile range.

**Table 2:** Rheumatoid factor and anti-cyclic citrullinated peptide antibody test results in 1110 Korean subjects.

Anti-CCP	At Initial Measurement			Overall Measurement		
	Rheumatoid Factor			Rheumatoid Factor		
	Negative	Positive	Total	Negative	Positive	Total
Not positive ^1^	737 (66.4%)	191 (17.2%)	928 (83.6%)	1660 (64.7%)	433 (16.9%)	2093 (81.6%)
Positive	22 (2.0%)	160 (14.4%)	182 (16.4%)	65 (2.5%)	408 (15.9%)	473 (18.4%)
Total	759 (68.4%)	351 (31.6%)	1110	1725 (67.2%)	841 (32.8%)	2566

Data from patients with follow-up samples. ^1^ Negative and equivocal results. Abbreviations: anti-CCP, anti-cyclic citrullinated peptide antibody.

## Data Availability

The datasets generated and analyzed during the current study are available from the corresponding authors upon reasonable request.
